# Long-Term Exposure to Ambient Particulate Matter and Structural Brain Changes in Older Adults

**DOI:** 10.1161/STROKEAHA.124.048096

**Published:** 2025-05-12

**Authors:** Giulia Grande, Bolin Wu, Jing Wu, Grégoria Kalpouzos, Erika J. Laukka, Tom Bellander, Debora Rizzuto

**Affiliations:** Aging Research Center, Department of Neurobiology, Care Sciences and Society, Karolinska Institutet and Stockholm University, Sweden (G.G., B.W., G.K., E.J.L., T.B., D.R.).; Stockholm Gerontology Research Centre, Sweden (G.G., E.J.L., D.R.).; Institute of Environmental Medicine (IMM), Karolinska Institutet, Sweden (J.W., T.B.).

**Keywords:** air pollution, atrophy, brain, stroke, white matter

## Abstract

**BACKGROUND:**

Accumulating evidence links air pollution exposure to late-life cognitive deterioration. Whether air pollution alters brain structure remains poorly understood. Thus, we aimed to quantify the association between long-term exposure to particulate matter ≤2.5 µm and ≤10 µm (PM_2.5_ and PM_10_, respectively) and late-life brain structural changes.

**METHODS:**

In the Swedish National Study on Aging and Care in Kungsholmen, Stockholm, 555 participants free from dementia underwent brain magnetic resonance imaging (MRI) scans at baseline and after 6 years (cohorts <78 years) or after 3 and 6 years (cohorts aged ≥78 years). After the exclusion of participants with neurological conditions (including previous stroke) and suboptimal MRI quality, we had 457 participants with available repeated MRI examinations, where total brain tissue volume, ventricles, hippocampus, and white matter hyperintensities volumes were assessed. PM_2.5_ and PM_10_ have been assessed since 1990 using dispersion models at residential addresses. Brain volumes have been standardized using baseline mean and SD. Long-term exposure to PM_2.5_ and PM_10_ in relation to the baseline and longitudinal brain MRI volumes were tested through multiadjusted (age, sex, educational level, smoking, socioeconomic status, and neighborhood household mean income) linear regression models.

**RESULTS:**

At study entry, the mean (SD) age of the participants was 70 (SD, 8.9) years and 41% were males. Individuals who before baseline had been exposed to levels of PM_2.5_ or PM_10_ above the median (8.5 and 14.9 μg/m^3^, respectively) had smaller total brain tissue volume (β, −0.20 [95% CI, −0.33 to −0.06] and β, −0.14 [95% CI, −0.28 to −0.01], respectively) at baseline than those with lower PM_2.5_ and PM_10_ levels. Participants exposed during the follow-up to PM_2.5_>8.7 μg/m^3^ had on average an annual shrinkage of total brain tissue volume of 0.22 (95% CI, −0.43 to −0.01) and an annual increase of 0.25 (95% CI, 0.07–0.43) of the white matter hyperintensities as compared with participants exposed to PM_2.5_<8.7 μg/m^3^. No association was detected between PM_10_ and an annual rate of change in brain MRI volumes.

**CONCLUSIONS:**

Long-term exposure to comparatively low levels of PM_2.5_ was associated with a greater load of structural brain changes, encompassing brain atrophy and vascular pathology. These findings, in a dementia- and cerebrovascular disease-free sample, underscore the importance of addressing air pollution as a modifiable risk factor for late-life cognitive decline, and highlight the need for targeted interventions to prevent its detrimental effects on brain integrity.

Exposure to ambient air pollution has recently emerged as a significant environmental risk factor for accelerated cognitive aging.^1^ Indeed, numerous studies have shown that long-term exposure to air pollution, especially fine particulate matter (PM) with aerodynamic diameter ≤2.5 µm (PM_2.5_) may increase the risk of dementia, including Alzheimer disease.^2,3^

However, despite the mounting evidence underscoring the harmful effects of PM_2.5_ and other pollutants on the brain, the underlying mechanisms remain elusive to date.^4^ Studies conducted in both animal and human subjects have provided evidence that associates air pollution exposure with faster deposition of amyloid-β and neurofibrillary tangles.^5^ Additionally, vascular pathology might also contribute to this complex relationship, with stroke being a significant intermediate factor between air pollution and the development of dementia.^6,7^ Although some studies have investigated the link between environmental exposures and markers of brain damage,^3,8–10^ such as smaller total brain tissue volume, decreased hippocampal size, and greater amount of white matter lesions^9,11^ most of these studies are based on cross-sectional study designs, thus warranting further investigation with longitudinal perspectives.

Therefore, we conducted a longitudinal study to examine the association between long-term exposure to PM_2.5_ and PM_10_ and pathological brain changes, using a well-characterized population-based study with spatially highly resolved data on long-term exposure to air pollution, clinical assessments, and repeated brain magnetic resonance imaging (MRI).

## Methods

### Study Population

The Swedish National Study on Aging and Care in Kungsholmen Brain Magnetic Resonance Imaging Study (SNAC-K-MRI) is part of the larger SNAC-K study. SNAC-K is an ongoing population-based longitudinal study that had baseline assessments between 2001 and 2004 and included overall 3363 (response rate, 73.3%), ≥60 years older adults who were residents of the Kungsholmen district in central Stockholm, as detailed elsewhere.^12^

The SNAC-K-MRI study includes a subsample of 555 noninstitutionalized, nondisabled, older adults without dementia who agreed to undergo structural brain 1.5 Tesla MRI scans.

Individuals <78 years (younger cohort) were reassessed and repeated the MRI scan after 6 years, whereas those 78+(older cohort) repeated the assessments after 3 and 6 years.

Of those who completed brain MRI, we excluded 98 participants with either neurological or neuropsychiatric conditions (including previous stroke, tumors, and aneurysms) and suboptimal MRI quality (ie, excessive head motion artifacts and technical issues during the scanning). Thus, the final baseline analytic sample comprised 457 participants of whom 296 had at least another follow-up assessment.

Ethical approval for this study was obtained from the regional ethical review board in Stockholm, Sweden. All participants provided written informed consent for each study visit.

The findings of this study adhere to the STROBE (Strengthening the Reporting of Observational Studies in Epidemiology) recommendations for observational research. Deidentified SNAC-K data that supported these findings will be made available upon request by qualified researchers at https://www.snac-k.se/ in compliance with institutional policies.

### Data Collection

Data collection was conducted at our research center following standardized procedures. Trained staff conducted face-to-face interviews, along with clinical and laboratory examinations.

Information about age, sex, and education was gathered through personal interviews administered by trained nurses. Education level was determined based on the participant’s highest level of formal education and classified as elementary, high school, or university and above. Dementia in SNAC-K is clinically diagnosed in keeping with the DSM-IV criteria (Diagnostic and Statistical Manual of Mental Disorders, Fourth Edition)^13^ and follows a standardized 3-step procedure.^6^ Socioeconomic status was derived from the participant’s longest-held occupation and categorized into groups: blue-collar workers, white-collar workers, and entrepreneurs. We calculated the neighborhood household mean income (equivalized disposable income) for each individual in the study as a proxy for socioeconomic status based on residential address. This information was obtained from Statistic Sweden and calculated based on DeSO, Demographic Statistical Areas,^14^ in line with the previously described methodology.^15^ Smoking data were categorized as current, former, or never smokers. Detailed information on the full address history for all individuals included in the study during the whole study period is available in SNAC-K. For this study, none of the participants included at baseline, moved outside the district of Kungsholmen during the entire follow-up time.

### Air Pollution Exposures

Based on each participant’s residential address, which was regularly updated from the tax registry, historical exposure to PM_2.5_ and PM_10_ was estimated based on ambient levels derived from spatio-temporal modeling, using emission inventories for the years 1990, 1995, 2000, 2005, and 2011.^16^ Dispersion modeling uses mathematical formulations to characterize the atmospheric processes that disperse a pollutant emitted by a source. Based on emissions and meteorologic inputs, a dispersion model can be used to predict concentrations at selected downwind receptor locations. Gaussian dispersion models with a quadtree receptor grid ranging from 35 to 500 m side squares were applied to the emissions together with climatologies. For the period 1990 to 2010, annual average levels of PM_2.5_ and PM_10_ were obtained from linear interpolation over the 4 years between each model simulation.

We created 2 sets of exposure windows to separately consider exposure before baseline and during follow-ups. First, we considered 5-year average exposures preceding the baseline assessment (2001–2004). In addition, we considered the average exposure between baseline and the last available follow-up examination, 2004 to 2007 or 2007 to 2010.

### Neuroimaging Data Acquisition and Processing

The baseline MRI acquisition protocol and details of the imaging processing are described in the Supplemental Text. In brief, T1-weighted images were segmented into gray matter volume, white matter volume, and cerebrospinal fluid volume using SPM12 in Matlab 10. Total brain tissue volume (TBTV) was calculated by summing the gray matter volume and white matter volume. Total intracranial volume was obtained by summing gray matter volume, white matter volume, and cerebrospinal fluid volume. FreeSurfer automated segmentation was used to extract the hippocampal volume. The lateral ventricles were segmented automatically in the ALVIN toolbox and their volumes were calculated.^17^ A neuroimaging expert (G.K.) visually inspected each of the segmentations and manually drew the white matter hyperintensities volume on fluid-attenuated inversion recovery images.

The MRI analyses were done blind to the clinical data. All MRI measurements were adjusted by total intracranial volume, and the adjusted volumes were used in data analyses. To allow comparability between coefficients, we standardized brain volumes using baseline mean and SD.

### Statistical Analyses

First, we performed linear regression analyses to quantify the association between PM_2.5_ and PM_10_ levels over the 5 years preceding the baseline assessment and MRI markers at baseline. The pollutants were analyzed both as continuous and as categorical variables, with a cutoff set at their medial levels (8.5 μg/m^3^ for PM_2.5_ and 14.9 μg/m^3^ for PM_10_). The models were adjusted for age, sex, educational level, smoking, socioeconomic status, and neighborhood household mean income.

Additionally, we examined the relationship between levels of PM_2.5_ and PM_10_ during the follow-up period, both as continuous and as categorical variables (above the median level, 8.7 μg/m^3^ for PM_2.5_ and 15.2 μg/m^3^ for PM_10_) and the annual rate of changes in MRI markers.

The annual rate of changes in MRI volumes was determined as follows:


$$\begin{array}{l} r\left(i\right)=\;\;\;\frac{\Delta i}{n} \end{array}$$

where Δi is the change between the baseline of the follow-up assessment of the biomarkers *i* (total brain volume, hippocampal volume, ventricles, and white matter hyperintensities volume); *n* is the number of years over which the change occurred. The first (2004–2007) or the second (2007–2010) follow-up assessment was considered, depending on data availability. These analyses were further adjusted for baseline brain volumes.

### Sensitivity Analysis

To assess whether the loss to follow-up might affect the results, a logistic regression estimated the probability of dropping out considering age, sex, education, number of chronic diseases, smoking, and air pollution levels at baseline, and an inverse probability weight was generated. These weights were incorporated into the regression models to address potential bias due to dropout data.

All analyses were performed using Stata (version 18.0; StataCorp).

## Results

At study entry, the mean (SD) age of the participants was 70 (SD, 8.9) years, 41% were males, and 88% had an education level of high school or above (Table). Older (≥78 years) and younger (<78 years) participants differed in education, smoking habits, and MRI volumes.

**Table.**
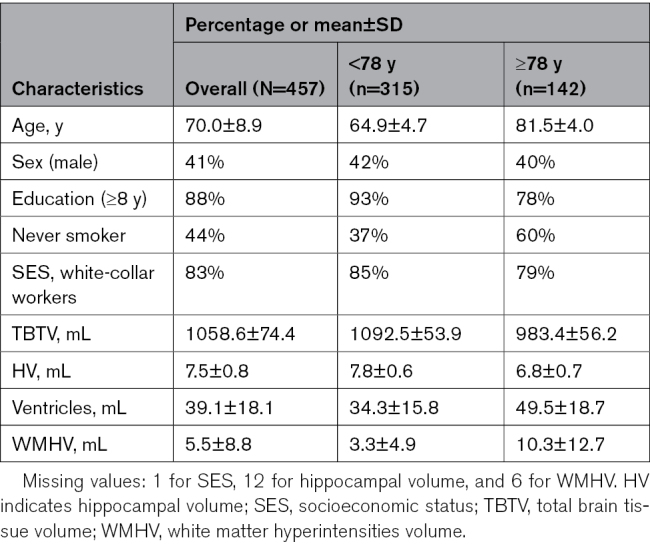
Baseline Population Characteristics Overall and by Age Groups

Overall, at baseline, there were 12 missing values for hippocampal volume, 6 for white matter hyperintensities, 1 for PM_2.5_, and 1 for socioeconomic status. We found no statistically significant associations between age, female sex, education, number of chronic diseases, and PM_2.5_ exposure level and being missing.

**Figure 1.**
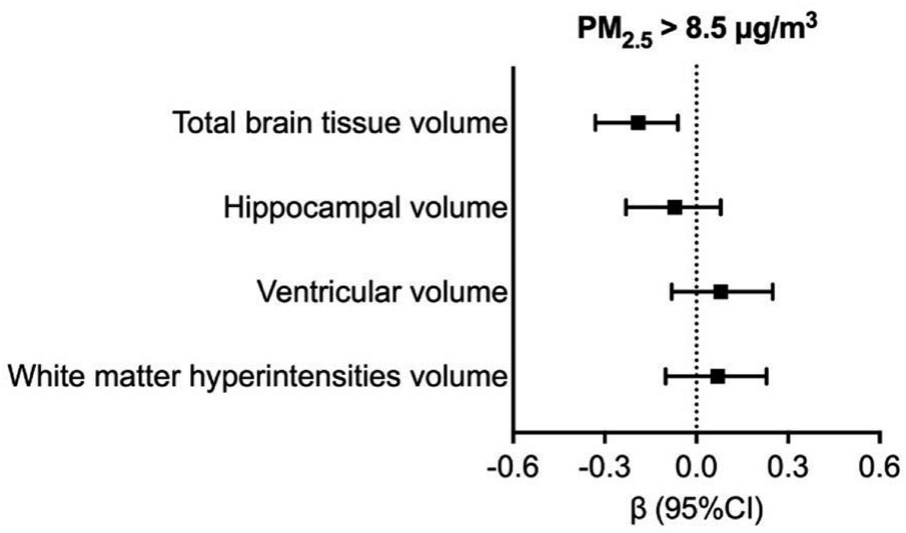
**Associations between exposure to particulate matter (PM_2.5_) during the 5 years preceding baseline and standardized magnetic resonance imaging markers at baseline.** β Coefficients with 95% CIs are derived from linear regression models, adjusted for age, sex, educational level, smoking, socioeconomic status, and neighborhood household mean income. All volumes were adjusted for total intracranial volume and converted into *z* scores.

At baseline, in multiadjusted linear regression models, we found a significant linear association between PM_2.5_, PM_10_, and TBTV, but no significant associations with other brain volumes (β, −0.10 [95% CI, −0.20 to −0.01] for PM_2.5_; β, −0.03 [95% CI,−0.06 to −0.01] for PM_10_). These results are reported in Table S1. Participants exposed to a level of PM_2.5_ above 8.5 μg/m^3^ (median) and of PM_10_ above 14.9 μg/m^3^ (median) during the 5 years before baseline had a standardized TBTV of −0.20 (95% CI, −0.33 to −0.06) and −0.14 (95% CI, −0.27 to −0.01) smaller respectively than people exposed to lower levels of PM_2.5_ and PM_10_ (below 8.5 and 14.9 μg/m^3^, respectively) (Figure 1; Table S2). No statistically significant associations arose between PM_2.5_ and PM_10_ and other brain volumes (ventricles, hippocampal volume, and white matter hyperintensities [WMHs]).The mean rate of change of standardized MRI parameters during on average 5.5 years of follow-up was −0.04 (95% CI, −0.14 to 0.07)/y for TBTV, −0.02 (95% CI, −0.14 to 0.09)/y for hippocampal volume, 0.01 (95% CI, −0.13 to 0.10)/y for ventricular volume, and 0.05 (95% CI, −0.04 to 0.15)/y for white matter hyperintensities volume.

During the follow-up period, we found no associations between continuous PM and brain MRI volume changes (Table S3).

**Figure 2.**
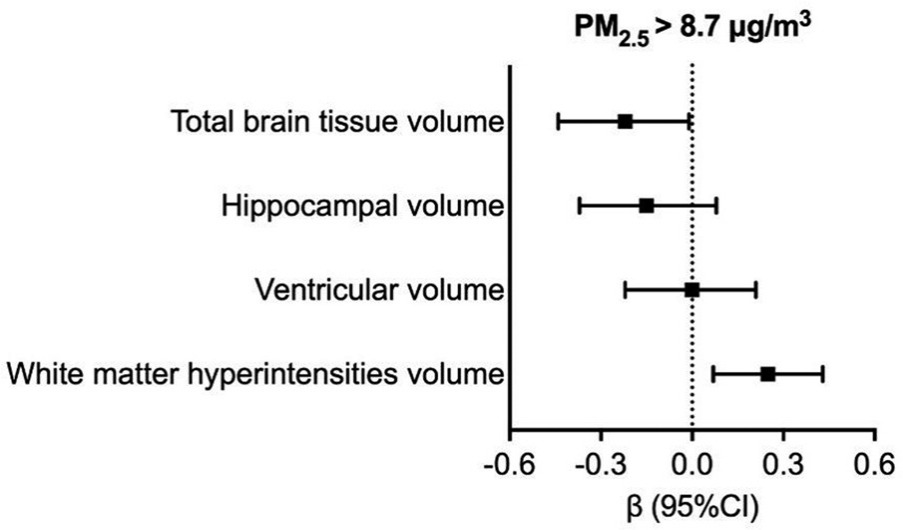
**Association between particulate matter (PM2.5) with annual rate of changes of magnetic resonance imaging (MRI) volumes over up to 6 years.** β Coefficients with 95% CIs are derived from linear regression models, adjusted for age, sex, educational level, smoking, socioeconomic status, neighborhood household mean income, and baseline measure of the specific MRI volume. All volumes are adjusted for total intracranial volume and converted into *z* scores.

Compared with participants exposed to PM_2.5_ below the median (8.7 μg/m^3^), higher exposed participants had a higher decrease of TBTV by 0.22 (95% CI, −0.44 to −0.01)/y, and a higher increase of WMH of 0.25 (95% CI, 0.07; 0.43)/y (Figure 2). No statistically significant associations were detected between PM_2.5_ and hippocampal volume (β,−0.15 [95% CI, −0.38 to 0.08]) or ventricles volume (β, 0.00 [95% CI, −0.22 to 0.21]). No statistically significant associations arose between PM_10_ when categorized around the median and brain volume changes (Table S4).

### Sensitivity Analysis

The results remained similar after including in the models the inverse probability weighting for dropout status (Figure S1).

## Discussion

Based on the findings from this study, performed in a population setting, older adults residing in areas with higher levels, as compared with those with lower levels, of PM_2.5_ and PM_10_ exhibited smaller total brain tissue volume. In addition, those exposed to higher levels of PM_2.5_ accumulated higher loads of vascular pathology over time, in comparison with those exposed to lower PM_2.5_ levels. No statistically significant results were found in the longitudinal analyses for PM_10_. Noteworthy, our participants were free from dementia and a history of cerebrovascular diseases at baseline, suggesting that exposure to air pollution might have negative effects on brain structure even before a diagnosis of dementia and in individuals without clinically evident cerebrovascular conditions. Moreover, the results were evident in a study area with historically low concentrations of air pollution, emphasizing the need for further actions to reduce air pollution in urban areas. While previous research has explored brain damage linked to exposure to air pollution using cross-sectional MRI data, our study incorporates longitudinal brain volume data, thus expanding the existing knowledge.

Air pollution exposure has been recently acknowledged as a risk factor for all-cause and Alzheimer disease dementia,^18^ but the biological mechanisms underlying this link are so far poorly understood. Only a few community-based studies^8–11^ have integrated clinical data with neuroimaging assessments and found air pollution has detrimental effects on brain structures and functioning. For instance, Wilker et al^10^ examined the association between residential long-term exposure to ambient air pollution and markers of brain aging using MRI data from the Framingham Offspring Study and found an association between PM_2.5_ exposure and reduced total brain volume alongside increased odds of covert brain infarcts. Similarly, Chen et al^8^ conducted a study including 1403 community-dwelling older women without dementia who were part of the Women’s Health Initiative Memory Study and found exposure to higher PM_2.5_ was significantly associated with smaller white matter, but not gray matter volumes. Our findings align with this evidence, demonstrating an association between PM_2.5_ exposure and the longitudinal accumulation of microvascular pathology, as evidenced by increased WMH volumes. Interestingly, we were instead not able to detect any statistically significant association between PM_2.5_ exposure and hippocampal volume, although point estimates for both the cross-sectional and longitudinal analyses point toward an association.

Taken together, our and previous findings underscore the possibility that certain pathways or specific brain areas could be particularly vulnerable to neurotoxic insult from air pollution, while others may not exhibit the same susceptibility, at least in the initial stages. For instance, the lack of an association between PM_2.5_ and hippocampal volume found in our study was also shown in previous reports^8,10^ and aligns with the current toxicological literature that indicates only limited evidence for neuronal death in animals who inhaled concentrated fine particles.^19^ However, this evidence contrasts with previous ones. For instance, a study involving clinically healthy dogs exposed to urban air pollutants like ozone and PM revealed brain lesions, particularly in the hippocampus and frontal cortex, accompanied by β-amyloid deposition, neurofibrillary tangles, and vascular and perivascular lesions.^20^ However, such observations have not been consistently replicated in humans, with only a few studies on older adults showing a similar association.^9,11^ Concerning the lack of association between PM_2.5_ and hippocampal volumes in our sample, some considerations need to be made. First, our sample size may not have been sufficient to detect subtle changes in hippocampal volume, and larger studies are needed to further address this specific point. Second, the use of a 1.5 T MRI scanner, which is considered suboptimal compared with more advanced imaging technologies, may have impacted the accuracy of hippocampal measurements. Higher field strength MRI scanners provide greater resolution and could reduce measurement noise, potentially revealing associations that were not detectable with the equipment used in our study. Third, the relatively short follow-up period of 6 years may not have been sufficient to observe significant hippocampal atrophy due to exposure to PM. Longer follow-up studies are necessary to capture the long-term effects of pollution on hippocampal volume. Another aspect that requires attention, and may seem counterintuitive at first glance, is the observed loss in total brain tissue volume without corresponding hippocampal volume loss. However, brain atrophy related to vascular pathology could be a contributing factor, as reported in prior studies.^21^ The association with global brain atrophy suggests that PM exposure might lead to broader neurotoxic effects, potentially mediated by vascular changes or systemic inflammation. This underscores the complex and multifaceted nature of brain damage induced by PM exposure. Further research, with larger cohorts, advanced imaging techniques, and longer follow-up periods, is needed to clarify the exact relationship between air pollution and brain health.

Our results on the accumulation of WMHs in relation to high levels of PM_2.5_ (above 8.5 μg/m^3^) are not surprising as a vascular pathway linking air pollution to dementia has been previously suggested. In fact, we and others have observed stroke being an important intermediate condition between air pollution and dementia.^6,7,22^ In addition, vascular risk factors, such as hypertension, diabetes, and obesity, are well-known contributors to brain atrophy and have been linked to faster WMH accumulation^23^ and could potentially either modulate or mediate the relationship between air pollution and brain health. Given their relevance, future studies should carefully account for these risk factors to fully understand the multifaceted mechanisms through which air pollution impacts the brain. To note, we were able to detect such an association longitudinally but not at baseline. A few potential explanations could account for this finding. First, our baseline study sample includes only individuals without dementia or cerebrovascular diseases and who were cognitively healthy. Consequently, individuals who were frailer and potentially more affected by higher historical PM_2.5_ exposure may not be represented in the baseline cohort, as they might have been excluded due to preexisting conditions, cognitive symptoms, or death. This selection bias may have contributed to the lack of association observed at baseline. Second, it is possible that cumulative exposure to PM_2.5_ during the follow-up period exerted additional effects on brain health, leading to detectable changes in WMHs. These changes may have occurred on top of the global brain atrophy previously observed, potentially amplifying the impact during longitudinal assessments. Future studies including larger samples and more advanced imaging techniques are needed to better elucidate the mechanisms involved. The link between environmental pollution and cardiovascular morbidity and mortality is well-established and underscores the impact of air pollution on the vascular system.^24^ Animal and human studies have shown the initiation and progression of atherosclerosis in various arteries, including those in the brain, due to exposure to urban air particulates.^25^ Moreover, air pollution has been shown to exacerbate white matter perfusion deficits and disrupt the permeability of the blood-brain barrier. These effects on the vascular endothelium can lead to increased vascular resistance and decreased cerebral blood flow and these mechanisms together could explain the observed association between PM_2.5_ and WMH accumulation found in the present study. Of note, the impact of air pollution on the central nervous system extends beyond vascular mechanisms, as it also triggers a systemic inflammatory response characterized by elevated proinflammatory molecules, contributing to neuroinflammation, but also platelet activation, and thromboembolic events.^26^ Overall, air pollution shows multifaceted environmental toxicity, and it is plausible to hypothesize that it affects the brain through multiple complementary pathways that act synergistically rather than in isolation. Future studies are needed to account for those multiple mechanisms that may link air pollution exposure to poor brain health.

A mention should also be made of our results on PM_10_. While we found an association with total brain tissue volume at baseline, these were not statistically significant in longitudinal analyses, although point estimates pointed toward the same direction as PM_2.5_. This finding is not entirely surprising, as PM_2.5_ is generally recognized as more harmful to health compared with PM_10_.^27^ The smaller size of PM_2.5_ particles enables them to penetrate deeper into the respiratory system and potentially enter systemic circulation, thereby posing a greater risk for adverse health effects, including neurotoxicity.

Our findings come from a large, well-established population-based cohort with available repeated brain MRI measurements and spatially detailed information on long-term exposure to air pollution. The following limitations should be considered, particularly about the modeling of pollutants. We examined the associations between PM and brain volumes by treating the pollutants both as continuous variables and as categories around the median level. While we detected an association between binary PM_2.5_ levels and brain volumes, no association was found when considering PM as a continuous variable. We were unable to further explore dose-response relationships using percentiles due to the limited number of observations, which reduced our statistical power. Future studies with larger sample sizes will be necessary to clarify the nature of this relationship between environmental exposure and brain pathology. However, it is important to note that when studying environmental exposures such as PM_2.5_, threshold effects are often observed, meaning that the relationship with health outcomes is not linear across all levels of exposure.^28,29^ By categorizing PM, we aimed to capture these potential threshold effects, which could be obscured in a continuous analysis. Additionally, using PM as a continuous variable in a relatively small sample may be more susceptible to the influence of extreme values (outliers) and non-normal distributions. Categorizing the variable helps mitigate these issues, allowing for a more robust and reliable analysis of potential effects. Another potential limitation of this study is that SNAC-K, and particularly SNAC-K-MRI, includes older adults living in central Stockholm who are relatively healthy and fit, have a high socioeconomic status, and are mainly born in Sweden. This might limit the generalizability of our results to other countries or populations. Furthermore, besides the brain MRI parameters considered in the current study, other neuroimaging markers (eg, microbleeds, amyloid deposition) are worthy of further investigation in this context, but they were not available in our population-based dataset. In addition, the geographic area included, the Kungsholmen district of Stockholm, is small and limits spatial contrasts in air pollutants.

In summary, our study revealed that older adults exposed to high levels of PM_2.5_ exhibited smaller total brain tissue volumes, more global atrophy, and a higher accumulation of vascular pathology over time. This was evident in individuals free from dementia and cerebrovascular diseases, suggesting that air pollution exposure may negatively impact brain structure even in the absence of clinically evident cerebrovascular conditions or a dementia diagnosis. Moreover, the results were evident in a study area with historically low concentrations of air pollution, which underscores the need for additional efforts to reduce air pollution in urban areas, even in regions with relatively strict air quality standards. Future research should explore the specific mechanisms linking air pollution to brain health and investigate the effects of environmental pollutants on other neuroimaging markers. Our findings have important implications for public health interventions aimed at mitigating the adverse effects of air pollution on cognitive health in aging populations.^30–32^

## Article Information

### Acknowledgments

The authors thank the Swedish National Study on Aging and Care in Kungsholmen (SNAC-K) participants and the SNAC-K group for their collaboration in data collection and management. The funders had no role in study design, data collection, data analyses, data interpretation, or writing of the report. Drs Grande and Rizzuto contributed to the conception and design of the study. Dr Rizzuto conducted the statistical analyses. Drs Grande and Wu conducted the literature search. All the authors contributed to the interpretation of the results. Dr Grande drafted the first version of the article. All the authors critically revised the article for important intellectual content. All the authors made a significant contribution to the research and the development of the article and approved the final version for publication.

### Sources of Funding

Data collection of the Swedish National Study on Aging and Care in Kungsholmen (SNAC-K) was supported by the Swedish Research Council (current grant: 2021-00178), the Swedish Ministry of Health and Social Affairs, and the participating County Councils and Municipalities. This study was supported by the Karolinska Institutet Fonder (grant FS-2020:0007), Demensfonden (to Dr Grande), and Foundation for Geriatric Diseases at Karolinska Institutet (to Dr Grande, grant FS-2023:0007).

### Disclosures

None.

### Supplemental Material

Supplemental Text

Tables S1–S4

Figure S1

STROBE Checklist

## References

[1] Underwood E. The polluted brain. Science. 2017;355:342–345. doi: 10.1126/science.355.6323.342 28126768 10.1126/science.355.6323.342

[2] Sommerlad A Liu KY. Air pollution and dementia. BMJ. 2023;381:655. doi: 10.1136/bmj.p655 37019447 10.1136/bmj.p655

[3] Ru M Brauer M Lamarque JF Shindell D. Exploration of the global burden of dementia attributable to PM2.5: what do we know based on current evidence? GeoHealth. 2021;5:e2020GH000356. doi: 10.1029/2020GH000356 10.1029/2020GH000356 PMC8143277 34084981

[4] The Lancet Neurology. Air pollution and brain health: an emerging issue. Lancet Neurol. 2018;17:103. doi: 10.1016/S1474-4422(17)30462-3 29413304 10.1016/S1474-4422(17)30462-3

[5] Iaccarino L La Joie R Lesman-Segev OH Lee E Hanna L Allen IE Hillner BE Siegel BA Whitmer RA Carrillo MC. Association between ambient air pollution and amyloid positron emission tomography positivity in older adults with cognitive impairment. JAMA Neurol. 2021;78:197–207. doi: 10.1001/jamaneurol.2020.3962 33252608 10.1001/jamaneurol.2020.3962 PMC7879238

[6] Grande G Ljungman PLS Eneroth K Bellander T Rizzuto D. Association between cardiovascular disease and long-term exposure to air pollution with the risk of dementia. JAMA Neurol. 2020;77:801–809. doi: 10.1001/jamaneurol.2019.4914 32227140 10.1001/jamaneurol.2019.4914 PMC7105952

[7] Ilango SD Chen H Hystad P van Donkelaar A Kwong JC Tu K Martin RV Benmarhnia T. The role of cardiovascular disease in the relationship between air pollution and incident dementia: a population-based cohort study. Int J Epidemiol. 2020;49:36–44. doi: 10.1093/ije/dyz154 31347651 10.1093/ije/dyz154 PMC7124495

[8] Chen JC Wang X Wellenius GA Serre ML Driscoll I Casanova R McArdle JJ Manson JE Chui HC Espeland MA. Ambient air pollution and neurotoxicity on brain structure: evidence from Women’s Health Initiative Memory Study. Ann Neurol. 2015;78:466–476. doi: 10.1002/ana.24460 26075655 10.1002/ana.24460 PMC4546504

[9] Power MC Adar SD Yanosky JD Weuve J. Exposure to air pollution as a potential contributor to cognitive function, cognitive decline, brain imaging, and dementia: a systematic review of epidemiologic research. Neurotoxicology. 2016;56:235–253. doi: 10.1016/j.neuro.2016.06.004 27328897 10.1016/j.neuro.2016.06.004 PMC5048530

[10] Wilker EH Preis SR Beiser AS Wolf PA Au R Kloog I Li W Schwartz J Koutrakis P DeCarli C. Long-term exposure to fine particulate matter, residential proximity to major roads and measures of brain structure. Stroke. 2015;46:1161–1166. doi: 10.1161/STROKEAHA.114.008348 25908455 10.1161/STROKEAHA.114.008348 PMC4414870

[11] Weuve J Bennett EE Ranker L Gianattasio KZ Pedde M Adar SD Yanosky JD Power MC. Exposure to air pollution in relation to risk of dementia and related outcomes: an updated systematic review of the epidemiological literature. Environ Health Perspect. 2021;129:96001. doi: 10.1289/EHP8716 34558969 10.1289/EHP8716 PMC8462495

[12] Lagergren M Fratiglioni L Hallberg IR Berglund J Elmstahl S Hagberg B Holst G Rennemark M Sjolund BM Thorslund M. A longitudinal study integrating population, care and social services data. The Swedish National study on Aging and Care (SNAC). Aging Clin Exp Res. 2004;16:158–168. doi: 10.1007/BF03324546 15195992 10.1007/BF03324546

[13] American Psychiatry Association. Diagnostic and Statistical Manual of Mental Disorders (DSM-IV). American Psychiatry Association; 1994.

[14] SCB. Demographic Statistical Areas, DeSO. Accessed May 13, 2025. https://www.scb.se/hitta-statistik/regional-statistik-och-kartor/regionala-indelningar/

[15] Pyko A Andersson N Eriksson C de Faire U Lind T Mitkovskaya N Ogren M Ostenson CG Pedersen NL Rizzuto D. Long-term transportation noise exposure and incidence of ischaemic heart disease and stroke: a cohort study. Occup Environ Med. 2019;76:201–207. doi: 10.1136/oemed-2018-105333 30804165 10.1136/oemed-2018-105333

[16] Segersson D Eneroth K Gidhagen L Johansson C Omstedt G Nylen AE Forsberg B. Health impact of PM10, PM2.5 and black carbon exposure due to different source sectors in Stockholm, Gothenburg and Umea, Sweden. Int J Environ Res Public Health. 2017;14:742. doi: 10.3390/ijerph14070742 28686215 10.3390/ijerph14070742 PMC5551180

[17] Kempton MJ Underwood TS Brunton S Stylios F Schmechtig A Ettinger U Smith MS Lovestone S Crum WR Frangou S. A comprehensive testing protocol for MRI neuroanatomical segmentation techniques: evaluation of a novel lateral ventricle segmentation method. Neuroimage. 2011;58:1051–1059. doi: 10.1016/j.neuroimage.2011.06.080 21835253 10.1016/j.neuroimage.2011.06.080 PMC3551263

[18] Livingston G Huntley J Sommerlad A Ames D Ballard C Banerjee S Brayne C Burns A Cohen-Mansfield J Cooper C. Dementia prevention, intervention, and care: 2020 report of the Lancet Commission. Lancet. 2020;396:413–446. doi: 10.1016/S0140-6736(20)30367-6 32738937 10.1016/S0140-6736(20)30367-6 PMC7392084

[19] Block ML Calderon-Garciduenas L. Air pollution: mechanisms of neuroinflammation and CNS disease. Trends Neurosci. 2009;32:506–516. doi: 10.1016/j.tins.2009.05.009 19716187 10.1016/j.tins.2009.05.009 PMC2743793

[20] Calderon-Garciduenas L Maronpot RR Torres-Jardon R Henriquez-Roldan C Schoonhoven R Acuna-Ayala H Villarreal-Calderon A Nakamura J Fernando R Reed W. DNA damage in nasal and brain tissues of canines exposed to air pollutants is associated with evidence of chronic brain inflammation and neurodegeneration. Toxicol Pathol. 2003;31:524–538. doi: 10.1080/01926230390226645 14692621 10.1080/01926230390226645

[21] De Guio F Duering M Fazekas F De Leeuw FE Greenberg SM Pantoni L Aghetti A Smith EE Wardlaw J Jouvent E. Brain atrophy in cerebral small vessel diseases: extent, consequences, technical limitations and perspectives: the HARNESS initiative. J Cereb Blood Flow Metab. 2020;40:231–245. doi: 10.1177/0271678X19888967 31744377 10.1177/0271678X19888967 PMC7370623

[22] Grande G Hooshmand B Vetrano DL Smith DA Refsum H Fratiglioni L Ljungman P Wu J Bellavia A Eneroth K. Association of long-term exposure to air pollution and dementia risk. Neurology. 2023;101:e1231–e1240. doi: 10.1212/WNL.0000000000207656 37442622 10.1212/WNL.0000000000207656 PMC10516275

[23] Cox SR Lyall DM Ritchie SJ Bastin ME Harris MA Buchanan CR Fawns-Ritchie C Barbu MC de Nooij L Reus LM. Associations between vascular risk factors and brain MRI indices in UK Biobank. Eur Heart J. 2019;40:2290–2300. doi: 10.1093/eurheartj/ehz100 30854560 10.1093/eurheartj/ehz100 PMC6642726

[24] Suwa T Hogg JC Quinlan KB Ohgami A Vincent R van Eeden SF. Particulate air pollution induces progression of atherosclerosis. J Am Coll Cardiol. 2002;39:935–942. doi: 10.1016/s0735-1097(02)01715-1 11897432 10.1016/s0735-1097(02)01715-1

[25] Huuskonen MT Liu Q Lamorie-Foote K Shkirkova K Connor M Patel A Montagne A Baertsch H Sioutas C Morgan TE. Air pollution particulate matter amplifies white matter vascular pathology and demyelination caused by hypoperfusion. Front Immunol. 2021;12:785519. doi: 10.3389/fimmu.2021.785519 34868068 10.3389/fimmu.2021.785519 PMC8635097

[26] Wellenius GA Boyle LD Coull BA Milberg WP Gryparis A Schwartz J Mittleman MA Lipsitz LA. Residential proximity to nearest major roadway and cognitive function in community-dwelling seniors: results from the MOBILIZE Boston Study. J Am Geriatr Soc. 2012;60:2075–2080. doi: 10.1111/j.1532-5415.2012.04195.x 23126566 10.1111/j.1532-5415.2012.04195.x PMC3498530

[27] Finlayson-Pitts BJ Wingen LM Perraud V Ezell MJ. Open questions on the chemical composition of airborne particles. Commun Chem. 2020;3:108. doi: 10.1038/s42004-020-00347-4 36703388 10.1038/s42004-020-00347-4 PMC9814933

[28] Steenland K Deddens JA. A practical guide to dose-response analyses and risk assessment in occupational epidemiology. Epidemiology. 2004;15:63–70. doi: 10.1097/01.ede.0000100287.45004.e7 14712148 10.1097/01.ede.0000100287.45004.e7

[29] Grande G Wu J Ljungman PLS Stafoggia M Bellander T Rizzuto D. Long-term exposure to PM2.5 and cognitive decline: a longitudinal population-based study. J Alzheimers Dis. 2021;80:591–599. doi: 10.3233/jad-200852 33579834 10.3233/JAD-200852

[30] Ashburner J Friston KJ. Unified segmentation. Neuroimage. 2005;26:839–851. doi: 10.1016/j.neuroimage.2005.02.018 15955494 10.1016/j.neuroimage.2005.02.018

[31] Fischl B Salat DH Busa E Albert M Dieterich M Haselgrove C van der Kouwe A Killiany R Kennedy D Klaveness S. Whole brain segmentation: automated labeling of neuroanatomical structures in the human brain. Neuron. 2002;33:341–355. doi: 10.1016/s0896-6273(02)00569-x 11832223 10.1016/s0896-6273(02)00569-x

[32] Kohncke Y Laukka EJ Brehmer Y Kalpouzos G Li TQ Fratiglioni L Backman L Lovden M. Three-year changes in leisure activities are associated with concurrent changes in white matter microstructure and perceptual speed in individuals aged 80 years and older. Neurobiol Aging. 2016;41:173–186. doi: 10.1016/j.neurobiolaging.2016.02.013 27103530 10.1016/j.neurobiolaging.2016.02.013

